# Traditional birth attendants (TBAs) as potential agents in promoting male involvement in maternity preparedness: insights from a rural community in Uganda

**DOI:** 10.1186/s12978-016-0147-7

**Published:** 2016-03-12

**Authors:** Emmanueil Benon Turinawe, Jude T. Rwemisisi, Laban K. Musinguzi, Marije de Groot, Denis Muhangi, Daniel H. de Vries, David K. Mafigiri, Achilles Katamba, Nadine Parker, Robert Pool

**Affiliations:** University of Amsterdam, Amsterdam, The Netherlands; Makerere University, Kampala, Uganda; Amsterdam Institute of Global Health Development, Amsterdam, The Netherlands

**Keywords:** Traditional birth attendants, Male involvement, Maternal healthcare, Uganda

## Abstract

**Background:**

Since the 1994 International Conference on Population and Development, male involvement in reproductive health issues has been advocated as a means to improve maternal and child health outcomes, but to date, health providers have failed to achieve successful male involvement in pregnancy care especially in rural and remote areas where majority of the underserved populations live. In an effort to enhance community participation in maternity care, TBAs were trained and equipped to ensure better care and quick referral. In 1997, after the advent of the World Health Organization’s Safe Motherhood initiative, the enthusiasm turned away from traditional birth attendants (TBAs). However, in many developing countries, and especially in rural areas, TBAs continue to play a significant role. This study explored the interaction between men and TBAs in shaping maternal healthcare in a rural Ugandan context.

**Methods:**

This study employed ethnographic methods including participant observation, which took place in the process of everyday life activities of the respondents within the community; 12 focus group discussions, and 12 in-depth interviews with community members and key informants. Participants in this study were purposively selected to include TBAs, men, opinion leaders like village chairmen, and other key informants who had knowledge about the configuration of maternity services in the community. Data analysis was done inductively through an iterative process in which transcribed data was read to identify themes and codes were assigned to those themes.

**Results:**

Contrary to the thinking that TBA services are utilized by women only, we found that men actively seek the services of TBAs and utilize them for their wives’ healthcare within the community. TBAs in turn sensitize men using both cultural and biomedical health knowledge, and become allies with women in influencing men to provide resources needed for maternity care.

**Conclusion:**

In this study area, men trust and have confidence in TBAs; closer collaboration with TBAs may provide a suitable platform through which communities can be sensitized and men actively brought on board in promoting maternal health services for women in rural communities.

## Background

Maternal mortality remains a significant public health problem in developing countries, especially in sub-Saharan Africa, where 87 % of global maternal mortality occurs [1]. These high rates are attributed to insufficient and poor-quality care during pregnancy and childbirth [2]. Though the number of deliveries taking place in health facilities has increased globally, about 40 million women still deliver without skilled care every year, about a third of whom are assisted by a TBA [3]. The World Health Organization (WHO) recommends one midwife for every 175 pregnant women but this standard is far from being achieved; in Uganda, where 1.5 million women give birth every year, there are approximately 15,000 well-trained midwives to assist in the childbirth process [4]. Services provided by some TBAs may be limited to social support, while others provide full antenatal, intrapartum and postnatal care mediated by their indigenous cultural knowledge, medicines and skills, usually acquired through apprenticeship to other TBAs [5]. Studies find that TBAs are valued because they are accessible at all hours, are affordable and are culturally acceptable since they usually share similar cultures and may or may not be relatives, or neighbours with their client communities [6–8].

It has been argued that women utilize TBAs’ services due to their powerlessness in relation to men and older women in most traditional gendered contexts [9–13]. Especially in rural communities of developing countries, pregnancy and childbirth continue to be seen as a women’s issue, yet men control the resources that facilitate decisions concerning sexual relations, family size and access to healthcare [14]. Until the 1990s, many state and NGO interventions in maternal health targeted only women, especially promoting women’s visits to health facilities for maternal and child health (MCH) services, ignoring that men’s behaviour equally contributes to poor health outcomes for women and children [15]. However, recognition that male involvement is critical for improving MCH has since increased [16, 17]. A number of studies have shown that men play important roles in family planning and other maternal health issues [18–23]. This recognition shaped the recommendations made at the 1994 International Conference on Population and Development, which urged interventions that take account of men’s shared responsibility in improving MCH [24]. Though male involvement in reproductive health issues has gained more visibility, especially due to efforts to prevent mother-to-child transmission of HIV, gendered beliefs still define roles and influence how maternal health issues are approached in many parts of the developing world [25–27].

As early as the 1950s, there was a recognition that cultural beliefs, norms and practices concerning pregnancy situate men and TBAs as powerful actors in maternal health issues across the developing world [21, 24]. Thus, international agencies led by the WHO had accepted that TBAs, through their indigenous knowledge practices, were part of the problem of maternal ill-health, and concluded that training them could be part of the solution. By the early 1960s, the WHO was actively encouraging developing countries to train TBAs and by the 1990s, 85 % of the developing countries had some sort of collaboration with trained TBAs [28]. Training and activities of TBAs, as well as their legal status, varied across regions, countries and over time [28, 29].

By 1997, enthusiasm for training TBAs started to wane due to a lack of evidence that such training demonstrably reduced maternal mortality, and the finding that TBAs were contributing to delays in seeking care since they could not respond to obstetric emergencies [30, 31]. Attention shifted to the promotion of ‘skilled birth attendants’—health workers with midwifery skills—including the ability to respond to obstetric emergencies; this however did not define other skills that TBAs might also have. Thus some writers argued that exclusion of the trained TBAs from the definition of a skilled birth attendant was based on biased evidence [6]. It was also argued that ways of measuring maternal mortality had changed over time, and therefore the trends that indicated persistently high rates of maternal mortality did not reflect the contributory effects of those TBAs who had been trained [3, 6, 7, 32–35].

In Uganda, the policy towards TBAs shifted according to the recommendation of the WHO and the Safe Motherhood initiative. Promotion of skilled birth attendants—whose definition excluded TBAs—became the official policy, leading to the suspension of previously existing partnerships between the government and TBAs across the country. The Ugandan government recommended terminating collaborations between NGOs and TBAs as well. It held that the trained TBAs would be included in the newly formed village health teams (VHTs) if their respective communities selected them [34, 36]. As part of this shift, the government stopped training, support and supervision of TBAs, and enforced a ban on their involvement with deliveries. TBA services continue to be vaguely regarded as a type of informal support; there is no clear guidance on how they should be incorporated into the VHTs or on any legal implications for practice. VHTs are supposed to be elected by popular vote by local communities, but many TBAs have not been elected [37]. Despite such policies that hinder or ban the utilization of TBAs, it is reported that TBAs remain a popular source of care, attending to approximately 47–52 % of all deliveries in some remote districts of Uganda [38], due in part to the inability of government to enforce the ban, but also due to embeddedness of TBAs in community cultural practice. It has been argued that the generally poor functionality of the health system means that TBAs will continue to remain a source of care for many especially in rural areas of Uganda [16, 39–42].

It is widely acknowledged that cultural beliefs and norms position both men and TBAs as powerful actors in maternal health in many communities [43, 44]. In this paper, we argue that promoting men’s involvement while discouraging TBAs’ is a contradiction and does not recognize the various communities’ cultural beliefs and health resource practices. Since both men and TBAs are powerful actors in most communities and thus are potential allies in increasing birth preparedness [45, 46], policies that target one and not the other underestimate the power of informal, community-level networks that can be harnessed to promote positive health outcomes. We conclude that in especially gendered patriarchal communities, TBAs and men occupy powerful positions and are strong actors who influence their communities regarding all aspects of life, and thus their interaction should not escape the analysis of maternal health at the community level [47].

The question that emerges is how the interaction between men and TBAs shapes the provision of maternal healthcare in such rural contexts. Using ethnographic fieldwork we explored this interaction in Uganda’s rural district of Luwero. We conclude that this interaction between men and TBAs is beneficial as it increases the involvement of men in maternal health services, and thus has implications for policies regarding maternal health in Uganda and similar communities in Africa and beyond.

## Methods

### Setting

The study was carried out in Luwero, a sub-county in central Uganda with an estimated population of 29,904 [38]. Luwero is an ethnically mixed district, with the Baganda constituting about 76 % of the district population followed by Baluli and Nubians at 3 % each. The minority ethnic groups have assimilated to the Ganda culture, and especially language. The predominant religious group is the Anglicans at 37 % followed by Catholics at 32 % and Moslems at 22 % the other being Pentecostal sects and Orthodox church. Though Christian and Islamic religion has become the main stream worship, African traditional beliefs and practices are still prevalent among most communities. Both systems of beliefs inform practice of rituals concerning, birth, death, marriage, and funeral rites. People belong to cultural units called clans, which form cultural organizations to which every member of community must belong. Many people have settled along clan and family units and therefore, many live in proximate distances with people with whom they share blood relationship. Marriage among people of the same clan is culturally prohibited, and therefore, many are related through marriage to the members of the surrounding clans. Being mainly rural, the population is mainly, engaged in peasant agriculture and roadside petty trade in agricultural and household items. The district is served by three main towns, Kasana-Luwero, Bombo and Wobulenzi, with a number of small trading centres along the main arterial highway that connects the capital with the northern region and South Sudan. According to the tiered national health delivery system of Uganda [37], a district is supposed to have a general hospital, followed by health centre IV at county level, health centre IIIs at sub-county, health centre II at parish level and village health teams (VHTs) working as a health centre I at the bottom majorly for referral purposes. This hierarchy reflects the level of equipment and services that are accessed. Full maternity services apart from emergency obstetric care are available at health centre III. Luwero district does not have a district general hospital but rather a health centre IV, and Luwero sub-county where the ethnography was done does not have a health centre III and II. Residents access treatment at the nearest health centre IV which doubles as the main district health facility, about 4 miles away from most of the catchment villages with transport costs averaging about Ugx, 3000 (1$) by motorcycle taxis which are the most available modes of public transportation. The only general district hospital that is equipped to provide full services including emergency obstetric care is approximately 20 miles from the community. In 2012, only 52 % of deliveries were attended by a skilled birth attendant [48].

### Study design

This study was carried out as part of a broader study entitled Development of Sustainable Community Health Resources (CoHeRe) in resource-poor communities of Uganda under a partnership between Makerere University and University of Amsterdam. It involved extended ethnographic fieldwork between February 2013 and March 2014. Three authors (EBT, LKM and JTR) lived in the area for an extended period and collected the ethnographic data. The rest participated in other data collection and analysis activities including, field notes during spot checks, member checks, FGDs, dissemination and feedback activities, and supervision of data collection. Initially, purposive sampling was employed, targeting people and events that would give the researchers as much insight as possible. We observed daily life in the community and special events like community-wide meetings, weddings, burials and funeral rites. While observing such activities, data was collected through random and spontaneous conversations that were then analysed to identify recurrent issues and concepts; these comprised themes for further investigation. When the theme of TBAs as helpers in the event of pregnancy and childbirth emerged, we investigated it further through other ethnographic data collection methods. Twelve focus group discussions were held in the community: 6 with women, 3 with men and 2 with male VHT members. Twelve in-depth interviews were conducted: 5 with TBAs, 3 with VHT members, 2 with men, and 2 with district health officials. These were complemented with field notes taken during observational activities in the community on everyday life activities, and also with field notes taken by the researchers during visits to the antenatal clinic. All respondents were purposively selected to represent certain segments of the population—namely, men, women, youths, community leaders, TBAs and members of the village health teams.

### Ethical considerations

All interviews were tape-recorded after seeking and gaining permission of the respondents. The researchers were explicitly introduced as such to all community members at the beginning of the fieldwork period so that all people understood that the community was being studied for academic purposes. The social situations observed did not expose the studied community to any adverse effects. All collected data was secured under password-protected files that were accessible only to the research group. Pseudonyms have been used in reporting on this study in order to ensure confidentiality. Study approval was obtained from the ethical advisory board at the University of Amsterdam and Makerere University School of School of Public Health Higher Degrees, Research and Ethics Committee and registered by the Uganda National Council for Science and Technology [protocol number SS3420].

### Data analysis

Data analysis was done concurrently with data collection in order to identify and rectify errors during interviews and focus group discussions. Detailed field notes were discussed by the research team in order to identified points to subject to member checks with the respondents. Researchers carried out the member checks through interviews with respondents in order to ensure narrative accuracy and interpretive validity. In addition, dissemination workshop by the research team was organized at the end of fieldwork where pre-liminary analysis was shared and respondents gave feedback. After field work, all taped data were transcribed into English and together with field notes were entered into Nvivo 10 software for analysis. Of these raw data consisting of 42 sources, a query was conducted using the search terms “TBA”, “TBAs”, “Mulerwa”. This query led to a total of identified usages of any of these terms within 12 focus group discussions, 12 in depth interviews, 10 field notes. For each of these 34 documents the broad context of these search terms were researched and coded using an inductive coding strategy. Coding trees were created roughly falling with the following coding structure: a) TBAs appreciation of biomedical superiority in their practice, b) TBAs ban and stealth practice c) myths about TBA practice and pregnancy, d) TBA negotiation for women’s access to pregnancy services, e) TBA sensitization of men on pregnancy related services, f) Men’s trust of TBAs, g) TBAs roles in men’s birth preparedness. These codes were then analyzed to create the narrative structure of the case study which the research team discussed and came to agreement in cognizance of the respondents feedback and the experience of the researchers in the field. Data included direct quotes from participants; these were edited for grammar without altering original meanings.

## Results

### TBAs’ stealthy practice and appreciation of their limitations

Locally, a TBA is called a *Mulerwa* (*Bamulerwa*, plural), the word for an indigenous specialist in pregnancy and childbirth-related conditions. Their skills are learnt from their mothers or other older women in the village; the present-day biomedical health structure largely considers them inferior. Because they are not licensed to practice by the government, they are wary about talking about their work with strangers. In our first meeting with Juliet, a female TBA of about 60 years, she expressed uneasiness about talking to strangers:

I hope you are not a spy. You know, we don’t have [formal] qualifications like midwives. We were trained in handling pregnant women and managing deliveries. After the training, they used to give us gloves, safety blades, boots, lamps, polythene papers. The government has since stopped giving us those things, because we do not have the standards of hospitals. We are hiding in our homes [because the government stopped recognizing us as legitimate]. [But] people know our value and they find us here.

TBAs know that the government does not allow them to practice so they tend to keep their work underground. Because they know some conditions are beyond their capacity to manage, they tend to refer their clients to biomedical health facilities for antenatal care. For example, Juliet, a 60-year-old TBA said:

When the women come to me, I ask if they have attended antenatal care. Sometimes, men tell their wives to come to me instead of the health centre. But, here, I don’t have tetanus injections and I cannot test for HIV. I tell the women to go with their husbands and test for HIV before they come here. I cannot expose myself to the risk of HIV infection and besides, if she develops some complications that need [attention at the] hospital, they [medical staff] will demand proof of antenatal care.

TBAs encourage women to go to health facilities because there they can get services that TBAs cannot give. They try to get men to consider biomedical services that are related to safe pregnancy and childbirth. Similarly, Namulinde, a 68-year-old TBA, noted that when dealing with cases she suspects she cannot manage, she advises women to go to biomedical health care providers:

There is a young woman who came—it was her first pregnancy. She had gone to the health centre and they had confirmed the pregnancy. They had not told her though that she had twins, but when I checked her pregnancy I knew she had twins and asked her to make sure she delivers at the hospital [since it was her first time]. Another time I was called to deliver a girl who was very young. She was very fearful and I thought, ‘She is not well mentored; the baby might die during delivery’. I told them to proceed to the health centre, and from there, they were sent to a bigger hospital for an operation. I do not want be blamed if something goes wrong, so if I see that I cannot manage, I refuse the responsibility.

Both TBAs reported that they refer women to the medical centre if they encounter conditions that are difficult to manage. The knowledge about unmanageable cases may have been acquired from the training TBAs received in the late 1980s and early 1990s when they collaborated with biomedical workers. These expressions about their own practice reveal that TBAs learn a lot while working with formal health structures and though they operate almost underground, they do make referrals when they fear a woman’s condition may be beyond their expertise.

### TBAs ally with women to negotiate domestic power relations over maternity care

In our fieldwork, we learnt that sometimes women sought TBA services due to their husbands’ refusal or failure to provide permission and resources to enable them to access formal healthcare. However, TBAs, usually older women, ally with these women to negotiate domestic power relations, thus helping their clients to secure resources to access both formal and traditional healthcare. One TBA, Nanteza, narrated how she helped a young woman in the community, Sarah, to get money for a visit to the antenatal clinic from her husband:

Sarah’s husband works on a farm in Kiboga [60 km away]. He does not trust her and therefore does not want her to leave home. Before he left for his work, he asked me to help Sarah with all her pregnancy needs so that she would not have to go for antenatal care. He actually did not leave her with any money for antenatal care. When Sarah came to me, I advised her as usual to first go to the health centre for antenatal care, but she insisted she could not go because her husband did not permit her to travel and, besides, he did not leave her with any money for antenatal care. We had to work out a way together. I told the husband [on the phone] that he had to send his wife for antenatal care before I could start giving her local herbs. I asked him to send me 17,000 Uganda Shillings (5 US dollars) for the local herbs. I needed only 7000 for the local concoctions and the remainder was used by the wife for antenatal care. The husband returned after she had already visited the health centre.

This scenario illustrates how the TBA, recognizing the powerlessness of the younger woman, used her power as a respected and perhaps trusted woman to enable the younger woman to access the antenatal clinic.

In another interview, Nakanjako, a TBA of about 62 years, revealed how she navigates the power relations between men and women to secure maternal care for pregnant women, especially younger ones:

Some men are childish: the wife tells him about going to the health centre, he refuses. Sometimes they tell their wives to go to a *Mulerwa* instead of the health centre. For me, I demand *ekikuba nsiko* [a compensation usually charged in form of cash but can be in-kind]. Some women tell me to intervene because their husbands do not understand. Some men try to avoid me, but I look for them and insist that they pay for the services I give their wives. I also advise them to find time to have blood tests for HIV. Some men I confront end up changing their behaviour. I know what pregnancy means; I understand what the young women go through. I know the young husbands need counselling too.

TBAs use their power to help younger women navigate domestic power relations in accessing resources for pregnancy care. They become allies, and because of the biomedical training they have received, they pass on biomedical information in the process. Of course, their personal relationships with clients may play a motivating role, but then that is the nature of relationships in a small community. Neighbours may be related to one another by marriage or blood. For young women who rely on decisions by men to access pregnancy care, having older, respected allies is paramount.

### TBAs’ influence on men concerning maternity issues

TBAs actively sensitize men about both traditional and biomedical maternal issues when they interact in the process of giving care to women. When Nanteza told us how she helped Sarah get money from her husband, we inquired what the husband did when he learnt of the collusion between his wife and her. Nanteza told us:

He started to quarrel but I explained to him that the local herbs I give are not a replacement for other services from the health centre, for example, an HIV test. He was angry, reasoning that if his wife needed an HIV test, then she must be cheating on him. I calmed him down and showed him that she was doing the right thing because if she had HIV they can stop her from infecting the baby. He seemed unsure about what I told him, but he accepted it.

The TBA did not discredit the cultural local prescriptions, but sought to sensitize the man about the importance of an HIV test by relating it to the health of the unborn baby. In the same interview, she mentioned similar situations in which she has sensitized men:

Some men in this village still don’t understand why women should go for antenatal care. They think it is a way to avoid locally prescribed medicines that are part of the local culture. Some think lazy women go to the antenatal clinic to avoid work at home. If the woman has had a safe delivery before and next time there is a complication that leads to a stillbirth, she is blamed. I tell them that sometimes the body [uterus] of a woman is tired and not strong enough to hold another child. That is why *Bazungu* [Europeans] promote family planning. Some men want to blame these fatalities on their wives, accusing them of not taking local prescriptions. I tell such men to ensure enough spacing between children to allow the bodies of their women some rest. When I tell them, they go shaking their heads as if they didn’t know!

Though the biomedical essence of family planning is not to ‘give rest’ to the body, the TBA uses it metaphorically to sensitize men about the need for family planning and child spacing, a message that has been long left to biomedical staff, and have largely been unable to reach these same men in local communities. Another TBA, Nakati, about 62 years old, when asked how she relates to men, replied:

When I talk to them, especially those who come with their wives, I tell them to go and have an HIV test first. Some start by telling me that they do not have it but I insist, telling them, ‘If you do not have it, it doesn’t mean that your wife does not have it’. I tell them the advantage of the test, especially that if they have it, the unborn child can be kept safe. For some, I later learn from their wives that they did not go, but I make sure that the women have gotten tested because they are the ones whose bodies I touch [during childbirth].

As local, indigenous experts on maternity issues, TBAs are actively involved in passing on new information concerning family planning and prevention of mother-to-child transmission of HIV, knowledge that they have acquired from their interaction with the biomedical system. In their conversations with men, they counsel with practical information. It is evident that they do not only talk to men about their indigenous knowledge but also biomedical information. In rural settings where power asymmetries hinder women from introducing such talk in conversations with men, TBAs talking to men about HIV has a great deal of influence.

## Men trust TBAs’ advice and are willing to comply

Our ethnographic data show that men feel free to interact with TBAs concerning their wives’ maternal health issues especially during pregnancy and at childbirth. They trust and have confidence in the TBAs. In making decisions about the care of their wives and other women during pregnancy, men show a willingness to try and follow what TBAs tell them. Nanteza told me that men send their wives to her:

You find that women tell their men, ‘It is time to go to the clinic’. But the men insist and tell them, ‘Why don’t you go to Nanteza? Didn’t our mothers deliver us from Nanteza’s place? Did they have to go to the clinics?’

The men play an active role in encouraging the women under their influence to seek the services of TBAs in the community. In a focus group held with married/partnered men who had interacted with TBAs concerning maternity care, the discussion about a local TBA testified to this confidence and trust:

- Man 1: Even if your wife has never produced [given birth], the TBA can tell you if she is able to help her or not.
- Man 2: When you take your wife to her, she can check her pregnancy and give her our local medicines, which make her strong, and she can even induce birth pains when it is time. If that medicine fails to work within the estimated time, she can decide to send her to the health centre before the situation becomes worse.
- Man 3: She first checks, especially for the first timers. If she finds that some complications may occur, she sends her away, but for older women, she always tries her best.

These quotations show the confidence men have in TBAs and the trust they have in TBAs’ ability to assess the situation and determine when they cannot manage. In the quote above, men use active language when they refer to taking their wives to the TBA, but when it involves the woman to the health centre, they use passive language that the women ‘is sent’.This implies their level of involvement when care is accessed from the different sources.

Similarly, during another focus group men demonstrated the confidence they have in the TBA [Nakati] in their community:

- Man 4: This woman is better than the midwives. If you take your wife to the health centre, the nurses don’t give her any care. They just touch the stomach and thereafter they send her back with instructions to come back maybe after three months. Nakati gives the women local medicine—some for drinking and others for bathing. She can even detect the exact stage of the pregnancy and, besides, she will attend to her any time she goes there.
- Man 3: Nakati is twice as good as they [nurses] are. The only reason why we have to go to the health centre is to get the antenatal visit card so that in case of emergency complications they can help.

The men also showed some appreciation for antenatal care because the TBAs have been emphasizing it. The men seem to understand how TBAs work and believe that women should follow TBAs’ recommendations, as evidenced in the response from a focus group participant in Kyetume, who said:

It is important to go to her [the TBA] because she gives medicine to the pregnant woman to prepare her for delivery. For example, when the pregnancy is around seven months, she gives them herbs that *kumenya amagumba* (widen the pelvic bones). Sometimes the herbs can be mixed in *Emumbwa* [concoction dried in clay soil]. She does not help a pregnant woman who goes here for the delivery because she cannot be sure she took the right precautions to make the delivery safe.

Men have come to know what TBAs do in order to prepare women for safe delivery and they appreciate it. That is why men encourage women to visit TBAs, and especially so that women will be able to deliver under TBAs’ care later. TBAs seem to know the trust the men put in them and they use that to negotiate with men and to encourage them to participate in birth preparedness.

One man, Kitadda, about 36 years old, mentioned during a focus group that his wife had delivered twins with the assistance of a TBA. After the focus group, we approached him individually to get his comments on the experience. In the conversation, he revealed how the TBA involved him in preparing for the birth of the twins:

The TBA warned me to set aside money for transportation for my wife and other things in case of emergency. I gave my pregnant wife 22,000 Ugandan shillings [6 US dollars] which she kept. I gave her extra money to buy clothes and a polythene bag to use during delivery. The TBA warned me not to travel far or drink a lot of alcohol just in case she needed me. She warned that if I did not cooperate she would not help my wife. She gave me advice about saving food and also to make sure my wife did not get pregnant again soon after having twins. She also told me how to feed my wife so that she can produce enough breast milk for the twins. I knew she did not want to have trouble in case I was not there to make decisions in case of any emergency during the delivery process. She insisted I had to be there when my wife was delivering. I am the one she sent for some herbs to quicken labour pains.

The man prepared for the birth of the babies as demanded by the TBA. The TBA also counselled them on the different preparations he needed to undertake to care for the infants and the mother postpartum. This kind of relationship between men and TBAs illustrates the trust that exists between them.

### TBAs and men: the views of village health teams in the community

At the time of our fieldwork, village health teams (VHTs) were newly introduced community structures that had been created as a link between communities and formal healthcare. We sought the VHT members’ views on the relationship between TBAs and men. In a focus group, VHT members emphasized that they discourage the use of TBAs due to complications that the TBAs are not equipped to handle. One VHT member weighed in to explain why TBAs continue, however, to be popular:

We encourage all women to go to the health centre. And some trained TBAs send women with complications to the hospital. But if a woman goes to hospital, even if she delivers in the compound unattended, the husband comes and pays hospital fees. That is why the men tell the women, ‘Remain here and deliver from home’. Then next time he will take her to the TBA. But it is a risk.

In this quote, the VHT member infers that husbands are not present with wives when they deliver at the health centre. Interestingly, when referring to the TBA, he inferred that men take their wives. To this, he responded:

A TBA is known in the village … [she] can be a friend, or even a relative. The men go to her and negotiate for help when the wife is pregnant. Sometimes the men seek advice from the TBAs concerning the timing of the delivery. If it is about time, the TBA will ask for the necessary things. But at hospital, the nurses shout, ‘Where is the husband of this woman? Go and bring this, and that, do this, and the men feel vulnerable’.

The men are familiar with the environment in which TBAs work, and they do not feel vulnerable due to any power asymmetry between them and the practitioners. TBAs do not guarantee that there will be no complications but they sensitize all parties involved to take necessary precautionary measures.

## Discussion

As maternal mortality and morbidity remain a big challenge for low- and middle-income countries, there is a general agreement that increasing men’s involvement in maternal health issues will have a positive outcome on the health of women and children [17]. The majority of the factors that hinder male involvement in maternal health issues stems from sociocultural norms, beliefs and practices concerning reproduction [49]. Therefore, increasing male involvement may require a holistic approach, one that focuses on the community that shapes and is shaped by the actions of its members through their everyday life activities and local customs [50]. TBAs, in communities where they exist, occupy a social position which strategically positions them to become awareness creators and agents of behaviour change concerning maternity including bringing men on board.

In this paper, we have shown how the traditional role of a TBA is respected and is used to navigate maternity care issues..We observed that from previous collaboration between TBAs and the formal healthcare system, TBAs were able to appreciate modern medical practices and the superiority of modern medicine in managing challenges like HIV and other complications that may arise during pregnancy and childbirth. This knowledge that TBAs gained from training has continued to influence their informal practice and is the basis of their sensitization of men, even where there is no collaboration with formal healthcare to motivate their practice. This corroborates earlier studies that showed that training had increased TBAs’ knowledge and changed their practice [15]. It has been asserted that the TBAs’ willingness to attend training was motivated by their desire to gain more knowledge and improve their status in the community [51]. TBAs’ ability to provide advice on issues related to maternity care like antenatal visits, HIV testing and child spacing earned them more authority in this community and thus brought them more respect and trust, especially from men.

One of the barriers to timely access to maternal healthcare is the delay or refusal by men to commit resources [16]. Surprisingly, our findings show that because TBAs both command respect and understand the needs and challenges of women, they can be powerful allies for women in navigating domestic power relations and negotiating resources to access healthcare that may minimize the risk of complications. Indeed, the United Nations advised that achieving the Millennium Development Goals what needed to be done was as much social as it is medical, and thus recommends greater levels of men’s involvement in sexual and reproductive health issues at the community level [52]. Interestingly, we found that TBAs counselled men about maternal health issues. Men showed trust in and respect to TBAs when the latter engaged them especially on issues of HIV, family planning and child care during their interaction. These interactions are of great importance in rural communities where poor communication between men and women adversely affects women’s reproductive health and the health of infants, as men engage in risky behaviours out of ignorance [53]. It should therefore be encouraging to those who advocate greater male involvement to know that TBAs already voluntarily sensitize men on issues that health workers should, but fail to access them since many men do not visit health facilities.

Notably, TBAs work to involve men in birth preparedness and complication readiness. It has been argued that birth preparedness is key in saving women’s lives during childbirth yet it is very low in many rural areas of Uganda [16, 54]. As a condition for offering their services to women in the community, TBAs usually demand that husbands are prepared in case of emergencies that are too difficult to manage locally. Men usually comply by being on standby with the necessary resources in case the wife needs evacuation. TBAs thus have made men more conscious of childbirth emergencies, and involved them in planning for them. It has already been noted that trained TBAs are skilled in preparing their clients for birth by sensitizing them about emergencies [13, 51]. What is new here is our finding that the level of involvement by men is high through their cooperation with TBAs. Such birth preparedness, with men involved, increases the chances of a better health outcome for mothers through reduction of the delay in access to health facilities [17, 27, 49].

We have shown that TBAs are increasing male involvement in rural communities and offering relevant biomedical information even without officially collaborating with the formal health system [51]. Harnessing traditional institutions to offer new services is not a completely novel idea. In Uganda, a study identified the role of the *senga* (paternal aunt), who traditionally instructs adolescent girls about marriage and sex [55]. In that study, *senga*s were trained to provide advice on sexual and reproductive health, distribute condoms and link girls to the formal health sector where necessary. In their modified roles, the *senga*s became popular, attracting new clients including adult women and men. Similarly, the findings in this study lead us to believe that TBAs’ role in the community concerning male involvement in birth preparedness can be that of attracting men to maternity healthcare and increasing their awareness on the same issues since they are well trusted and share similar social space and position. As a previous studyrecommended, in order to improve pregnancy outcomes, families and communities need to be mobilized through effective community-based strategies [56]. TBAs as trusted community resource persons can be brought on board in the quest for a culturally sensitive approach to maternity care services in the especially in rural areas. This would be a stop-gap measure in the short and medium terms as poor staff attitudes, long distances, transport and delivery costs, are addressed to improve formal health coverage in the long run.

## Study limitation

The study covered one rural community in central Uganda and thus, while the results will provide important insights into the interaction between men and TBAs in ensuring women’s access to pregnancy and child delivery services, they remain limited in their generalizability. Also, because of the government policy that banned collaborations with TBAs, and since they knew that they were being studied, the respondents could have provided socially desirable answers. It should be noted however, that triangulation of data sources from different community members to verify single cases using different methods may validity of data. Also, long period which the researchers immersed in the everyday lives of the community provided sufficient time to build rapport with the respondents and may help to obtain honest and open responses due to reduced performativity from respondents. The studied community is fairly typical of many rural communities in Africa, and consequently, results are likely to be transferable to other similar settings in Uganda and other sub-Saharan communities.

## Conclusion

TBAs enjoy the community’s trust, which is conferred upon them by virtue of the culturally respected role they hold in society. This trust gives them authority in relation to their clients, including the women’s husbands. Due to TBAs’ earlier contact with the formal healthcare system during which they received training, they are able to sensitize their clients, including men, with biomedical information concerning maternal and child health. This information positively influences decisions for women’s and infants’ health. The shift of maternal healthcare policies away from collaboration with TBAs was informed by global recommendations [57], without taking into account local peculiarities in the communities served by TBAs. For many rural areas, TBAs are the only help accessible in the immediate neighbourhood [6, 28, 51]. Since TBAs acknowledge their shortcomings and are trusted in their communities by both men and women [8], their position is a culturally acceptable platform from which discuss and transform community norms concerning sexual and reproductive health [47, 58].

It has been claimed that one of the important maternal health issues that has not received adequate attention from donors and researchers is the role of unrecognized providers, among which TBAs are key, especially in rural areas today [59]. We hold that policy bans on the operation of TBAs is counterproductive; such policies are overly medicalised, and they fail to address the underlying reasons for TBAs’ existence. Such policy draws, in a top-down way, from a global menu of policy prescriptions that are insensitive to local context and thus tend to reproduce the inequities in health access that they seek to address. Trying to dissuade people from using TBA services while at the same pursuing policies that encourage male involvement is contradictory since it fails to recognize the sociocultural milieu within which the two interact in a symbiotic manner. Formal collaboration with TBAs could provide them with more training and equip them with biomedically approved skills, knowledge and health commodities that they would then disseminate to their clients in the communities. The examples provided in this article on the interaction between TBAs and communities, and especially men, concerning pregnancy-related services is evidence that this recommendation is viable.

The same may apply for other trusted, respected traditional roles that are embedded in communities where biomedical health professionals are in short supply. In such communities collaborations with people holding such roles may create culturally appropriate platforms for interventions that sensitize men and other community members about sexual and reproductive health.

## Abbreviations

MCH: Maternal and Child Health
NGOs: Non-Governmental Organizations
TBA: Traditional Birth Attendants
WHO: World Health Organization
